# Linkage Mapping and Molecular Diversity at the Flower Sex Locus in Wild and Cultivated Grapevine Reveal a Prominent SSR Haplotype in Hermaphrodite Plants

**DOI:** 10.1007/s12033-013-9657-5

**Published:** 2013-03-27

**Authors:** Juri Battilana, Silvia Lorenzi, Flavia M. Moreira, Paula Moreno-Sanz, Osvaldo Failla, Francesco Emanuelli, M. Stella Grando

**Affiliations:** 1 Department of Genomics and Biology of Fruit Crops, IASMA Research and Innovation Centre, Fondazione Edmund Mach Via E. Mach 1, 38010 San Michele all’Adige, TN Italy; 2 Department of Crop Production, Faculty of Agriculture, University of Milano, Via Celoria 2, 20133 Milan, Italy; 3 Present Address: Instituto Federal de Santa Catarina, Rua José Lino Kretzer 608–Praia Comprida, São José, Santa Catarina 88130-310 Brazil

**Keywords:** *V. vinifera*, Sex of the flower, Linkage mapping, Haplotype, Molecular breeding

## Abstract

Cultivars used for wine and table grape have self-fertile hermaphrodite flowers whereas wild European vines and American and Asian species are dioecious, having either male or female flowers. Consistent with previous studies, the flower sex trait was mapped as a single major locus on chromosome 2 based on a pure *Vitis vinifera* population segregating for hermaphrodite and female progeny, and a hybrid population producing all three flower sex types. The sex locus was placed between the same SSR and SNP markers on both genetic maps, although abnormal segregation hampered to fine map the genomic region. From a total of 55 possible haplotypes inferred for three SSR markers around the sex locus, in a population of 132 *V. sylvestris* accessions and 171 *V. vinifera* cultivars, one of them accounted for 66 % of the hermaphrodite individuals and may be the result of domestication. Specific size variants of the VVIB23 microsatellite sequence within the 3′-UTR of a putative YABBY1 gene were found to be statistically significantly associated with the sex alleles *M*, *H* and *f*; these markers can provide assistance in defining the status of wild grapevine germplasm.

**Electronic supplementary material:**

The online version of this article (doi:10.1007/s12033-013-9657-5) contains supplementary material, which is available to authorized users.

## Introduction

Flowers of *Vitis* spp. are typically unisexual. While male individuals possess erect functional anthers and lack a fully developed pistil, female flowers contain a functional pistil and either lack anthers or produce bent stamens and sterile pollen.

Selective vegetative propagation of mutant self-fertile Eurasian vines (*V. vinifera* L.) with bisexual flowers seems to have been crucial in developing domestic grapevine [1]. In fact, the majority of varieties cultivated worldwide today are hermaphrodites, which ensures greater yield and regular fruit production [2].

According to Antcliff [3] and Carbonneau [4], the model for sex inheritance in grape involves a single major locus with three different alleles, M (male), H (hermaphrodite) and F (female), in the following dominance relationship: M > H > F. Genetic linkage mappings are consistent with the single-gene hypothesis and the flower-type locus has been placed as a morphological trait on linkage group (LG) 2 of several genotypes [5–9].

Grapevine has a long generation time and inflorescence differentiation is not generally manifested in seedlings until they are 2–5 years old under cultivation or until they reach the forest canopy in the wild [10]. This is a hindrance in breeding programmes where evaluation of fruit set is essential for selecting and propagating desirable individuals. Molecular markers closely linked to the flower sex locus would be very useful for selecting out male types and for distinguishing self-fertile hermaphrodites from female phenotypes at the post-germination stage, thus saving time and resources in growing these plants in the field. However, although common microsatellite (SSR) loci relatively close to the sex locus have been reported, the general usefulness of these markers has not been tested across different germplasms. Recently, a molecular marker has been developed that identifies wild grape plants carrying a female sex allele [11] but male and hermaphroditic alleles are still to be differentiated.

The aim of this study is to provide further information about the sex locus by mapping the LG 2 based on a pure *vinifera* and a hybrid segregating population, and to test the diagnostic potential of markers linked to the type of flower. The gene content of the region associated with the flower sex was explored using the reference genome sequence and marker diversity around the sex locus was assessed in different *Vitis* germplasm. This allowed us to reconstruct probable haplotypes capturing the sex locus, to visualize their inter-connection and to check their frequency in wild and cultivated grapevine.

## Materials and Methods

A *V. vinifera* mapping population was created from a cross between hermaphrodite parents ‘Muscat Ottonel’ (Mo) and ‘Malvasia aromatica di Candia’ (Mc) and consisted of 91 F1 individuals. A second population was obtained from the interspecific cross between a hermaphrodite ‘Moscato bianco’ (*V. vinifera*) maternal parent (Mb) and a male grape accession (Vr) whose true origin was uncertain (*V. riparia* or *V. riparia* × *V. vinifera*). The latter progeny was previously used by Moreira et al. [12] to build complete linkage maps, and it was extended to 340 individuals for the present study.

Individuals of the mapping populations and germplasm accessions were scored for sex phenotype for 2 years during the flowering period by visual inspection using the descriptor OIV 151 (http://www.oiv.int/). In addition, two inflorescences per plant of the Mb × Vr population were protected from external pollination with white paper bags before flowering began. Fruit set was verified both outside and inside the bag at ripening time.

LG 2 was built using a panel of 15 SSR markers, whose position was known from previous studies [12–14], and 10 SNPs identified by direct sequencing of gene amplicons designed along chromosome 2 (http://www.genoscope.cns.fr/cgi-bin/ggb/vitis/gbrowse/vitis/?name=chr2). Primer sequences and nomenclature for SSR markers were obtained from multiple sources: the *Vitis* Microsatellite Consortium (VMC) managed by Agrogene SA (Moissy Cramayel, France), Bowers et al. [15] (VVMD), Sefc et al. [16] (VrZAG), Di Gaspero et al. [13] (UDV) and Merdinoglu et al. [17] (VVI). The primer sequences for SNP discovery and genotyping are reported in Table 1. Details of the procedure for DNA extraction, marker amplification, segregation analysis and map construction are given in Moreira et al. [12].

**Table 1:** List of primers used for SNP discovery and genotyping

Marker	Primer F	Primer R	SNP detection	Accession number	Gene prediction
1B_1	5′-GCATTTCTTCCCTCATCCAA-3′	5′-TTTCAGCAGAGGTGGGAGTT-3′	5′-TTAAAATCATCCAATGTTGC-3′	AM483743.2	LOC100241031
1B_2	5′-GCATTTCTTCCCTCATCCAA-3′	5′-TTTCAGCAGAGGTGGGAGTT-3′	5′-CTGGTTTCATCATTTTCAGG_3′	AM483743.2	LOC100247977
5C_2	5′-CCAATGTATAGGGGAATCAGGA-3′	5′-CACCAGCAAATTAGGCATATCA-3′	Resequencing	AM463701.1	LOC100241013
4C_1	5′-ATCTTGGGGTGTGGCATAAA-3′	5′-ATCATCCCACCTCTCACCAG-3′	Resequencing	AM485644.2	LOC100260606
4B_1	5′-TCAGATCTCTTCTGGCTTTTCTTT-3′	5′-CAAGCGTTTGGATTTTCCTC-3′	5′-GACAAGAAGAAAGATACAGA-3′	AM440935.2	LOC100258716
4B_2	5′-TCAGATCTCTTCTGGCTTTTCTTT-3′	5′-CAAGCGTTTGGATTTTCCTC-3′	5′-AAAGCAGGCCTCGGATTTTT-3′	AM440935.2	LOC100258716
2C	5′-TAAAGAGTTGGTGCGTGGTG-3′	5′-TTTGGGACTGTGAACCCTTC-3′	Resequencing	AM449705.1	LOC100251344
7C_1	5′-CAAAGGGCGGATTTAGTTTG-3′	5′-CAATGAGGGCAAGTCTGGTT-3′	5′-CTTTGTGCTTCTTCCCTAAC-3′	AM459636.1	–
7C_2	5′-ATCAACAGTTGTGGGCTTCC-3′	5′-GGGATGGTGAAGGAGTCGTA-3′	5′-GCATATTGCAGCAGATCAGC-3′	AM459636.1	LOC100266705
14A	5′-GATAATGGGCAGTTTGGACAAT-3′	5′-CTTACATTTCCTTGGGCAAAAC-3′	Resequencing	AM434019.1	–

Forward (F) and reverse (R) primers were used to amplify and sequence the genomic fragment on chromosome 2 for SNP discovery. Segregation analysis of the SNPs was performed based on minisequencing (SNaPshot) or resequencing the PCR product. NCBI http://www.ncbi.nlm.nih.gov/ ID of targeted loci on chromosome 2 (12× version of PN40024) are reported

Polymorphism at microsatellite loci in the sex genomic region was evaluated in 318 *Vitis* accessions structured into three genetically distinct subpopulations: 132 individuals of *V. vinifera* subsp. *sylvestris* (*V. sylvestris*), 171 cultivars of *V. vinifera* subsp. *vinifera* (*V. vinifera*) and 15 *Vitis* rootstock varieties bearing male-type flowers. All accessions are maintained in the FEM germplasm collection at San Michele all’Adige (Italy) and have distinct SSR genotypes at 22 loci.

The probable haplotypes were reconstructed using a Bayesian method implemented in the PHASE v 2.1 software [18]. Median-joining (MJ) Networks [19] were constructed with the Network 4.1.1.2 programme (Fluxus Technology Ltd, Clare, Suffolk, UK). The logistic regression model was applied to identify SSR polymorphisms and haplotypes associated with sexual determination using the General Linear Model (GLM binomial) function adapted to binary data and implemented in Rcmdr (R platform). Independent tests were carried out by comparing *vinifera* and *sylvestris* samples (Hermaphrodites = 1, non-hermaphrodites = 0), and specifically within the *sylvestris* population (female = 0, male = 1). An ANOVA test was used to check for type II errors when a false null hypothesis is not rejected.

## Results and Discussion

### Mapping and Characterization of the Genomic Region Associated with the Flower Sex

Flower sex is a serious obstacle to efficient grapevine breeding and improvement, given that up to 50 % of certain progeny populations (e.g. *ff* × *Hf*) have individuals with female flowers which are of no commercial use, since they require a male or hermaphrodite vine nearby to provide pollen to set the fruit.

Identical scores were obtained from visual inspections of the flower type and from the fruit set in the grapevines studied in this research survey. As expected, male individuals did not bear fruit, while hermaphrodites produced bunches both inside and outside the bags, and female vines produced fruits only outside the bags. In the pure *V.* *vinifera* mapping population (Mo × Mc), 75 % of the progeny was hermaphrodite, 23 % female and 2 % lacked inflorescences, showing that inheritance of flower type fits the parental genotype model *Hf* × *Hf*. On the other hand, in the hybrid population (Mb × Vr) three phenotypes were produced, 28 % hermaphrodite, 29 % female and 28 % male plants, while 15 % of the vines did not flower or could not be evaluated. Sex trait segregation was highly deviated (*P* < 0.0001) from the 1H:1F:2M ratio expected from *Hf* × *Mf* parents.

**Fig. 1:**
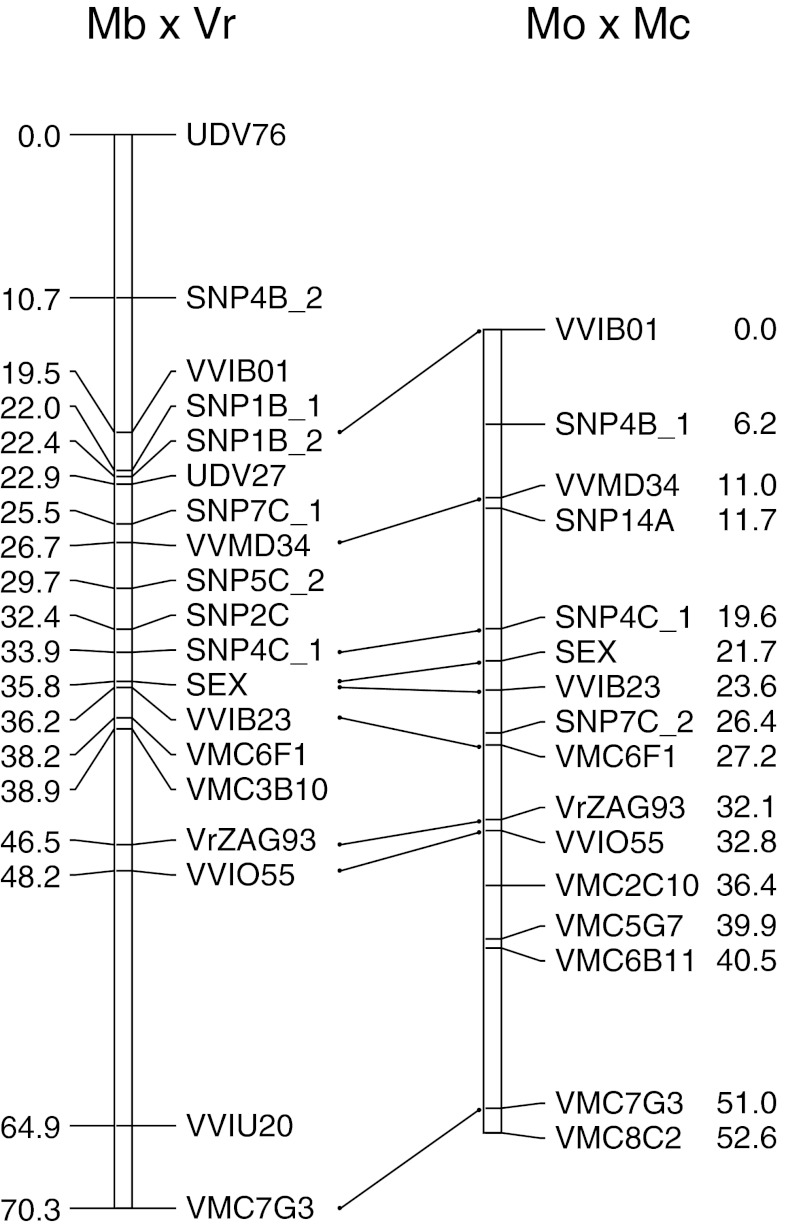
Genetic mapping of flower sex locus on Linkage group 2 (LG 2) built with datasets of SSR and SNP markers from two populations segregating for flower sex. LG 2 Mb × Vr was obtained by integrating individual maps of *V. vinifera* cv Moscato bianco and *V. riparia.* LG 2 Mo × Mc was obtained by integrating individual maps of *V. vinifera* cv Moscato Ottonel and *V. vinifera* cv Malvasia aromatica di Candia. Marker order was confirmed by aligning marker sequences on the physical map of PN40024

Linkage analysis was conducted on male and female datasets independently, then parental maps were merged into an integrated map. The LG 2 map obtained from the pure *V. vinifera* population spans 52.6 cM and includes 11 SSR and 4 SNP loci with an average marker space across the map of 3.3 cM (Fig. 1). All loci are collinear with the consensus LG 2 map of the hybrid population which spans 70.3 cM and integrates 11 SSR and 7 SNP loci with an average density of 3.7 cM.

The sex locus was mapped as a qualitative trait in both experiments based on segregation of the female phenotype in Mo × Mc and of the male phenotype in Mb × Vr. In the *V. vinifera* map the locus was 2.1 cM from marker SNP4C_1 and 1.9 cM from VVIB23, whereas in the hybrid population map the distance from the same markers were 1.9 and 0.4 cM, respectively. Beside a highly distorted segregation of markers from the paternal side in Mb × Vr, the shorter distance between the sex locus and VVIB23 was essentially due to the very low recombination rates exhibited by the male parent compared with the female parent (Supplementary Table 2). This may be related to the loss of male offspring and traced back to disturbed meiotic events and gametic or zygotic selection due, for instance, to incomplete pairing of homologous chromosomes in the sex genomic region as a consequence of the interspecific origin of the parents [20] or to a lethal recessive mutation as reported by Chaïb et al. [21] in other cultivars. For this reason further fine mapping of the sex region was precluded in Mb × Vr while no attempts were planned for Mo × Mc due to the limited size of the segregating population.

Our findings agree with previous experiments which reported VVIB23 and VVMD34 as being the closest markers to the sex locus [7, 8]. Marguerit et al. [9] placed the microsatellite VVIB23 at 4.5 cM from the sex locus in the female map and at 2.5 cM in the male map; it was also the nearest marker to the QTLs detected in LG 2 for inflorescence and flower morphology. Close linkage of VVIB23 to the trait was recently confirmed by Fechter et al. [11], although they placed the marker 1.0 cM away from the sex locus on the opposite side.

The SNP4C_1 and VVIB23 markers flanked a genomic region of 199 kbp located between 4,666,161 and 4,864,777 bp on the physical map of PN40024 (12× version) and 122 kbp upstream of the section fine mapped by Fechter et al. [11]. A list of 17 predicted genes was extracted from the reference grape genome (http://www.ncbi.nlm.nih.gov) and is reported in Supplementary Table 1. Several classes of genes were represented, including factors which might play a role in plant hormone metabolism and, consequently, have a potential impact on flower development. The list did not comprise the ethylene biosynthesis enzyme 1-aminocyclopropane-1-carboxylate synthase (ACS), which is an obvious candidate for the control of sex type in grapevine since Boualem et al. [22] identified differences in the ACS genomic sequence linked to sex phenotype in melon, *Cucumis melo*. However, it is remarkable that a gene encoding for ACS 10-like (LOC100244464) was predicted circa 200 kb upstream this section. This reinforces the suggestion of Fechter et al. [11] that the influence of this enzyme on sex formation in grapevine cannot be completely excluded. It was also observed that the microsatellite DNA sequence of VVIB23 is contained in the three prime untranslated region (3′-UTR) of a putative axial regulator YABBY1 (LOC100267708). Members of the YABBY gene family are expressed in a polar manner in all lateral organs produced by apical and flower meristems and are necessary for normal flower formation and development in *Arabidopsis* [23]. In table grapes, Costantini et al. [14] detected a significant QTL effect for flowering time and berry seed number associated to VVIB23 marker which may actually suggest a genetic influence of the locus on reproductive traits.

### Genetic Association Between Flower Sex and SSR Markers

Segregation analysis clearly indicated the VVIB23 marker size associated with each sex allele in the four parental plants (Table 2). The *f* allele was coupled with a 290 bp fragment both in ‘Muscat Ottonel’ and in ‘Malvasia di Candia’, while *H* alleles were associated with different microsatellite sizes in the two parents (284 and 288 bp, respectively). Hermaphrodite progeny from this cross were homozygous (*HH*) or heterozygous (*Hf*) at the sex locus. They exhibited only one recombinant genotype containing two copies of the 290 bp allele, although this low frequency may be due to the low resolution of the mapping experiment. The *f* and *H* alleles were associated with markers of the same size in ‘Moscato bianco’ and ‘Muscat Ottonel’, while the *M* and *f* alleles of the putative *V. riparia* parent were closely linked to 302 and 288 bp fragments, respectively. In Mb × Vr, the occurrence of recombination between the sex locus and VVIB23 locus was indicated by 35 progeny genotypes out of 270, of which only 5 were transmitted from the paternal side (Table 2 and Supplementary Table 2).

**Table 2:** Summary of phenotypic and genotypic variation in the flower sex trait and marker-trait association in the segregating populations

Plant material	Flower phenotype	Genotype at the sex locus	Allelic association between sex and VVIB23 marker (bp)
Muscat Ottonel (Mo)	H	*H*/*f*	H-284 f-290
Malvasia di Candia (Mc)	H	*H*/*f*	H-288 f-290
Mo × Mc population^a^	56H:18f	*H*/*H*, *H*/*f*, *f*/*f*	20HH:35Hf:19ff
Moscato bianco (Mb)	H	*H*/*f*	H-284 f-290
*V. riparia* (Vr)	M	*M*/*f*	M-302 f-288
Mb × Vr population^a^	88H:93M:89f	*H*/*f M*/*H M*/*f f*/*f*	96Hf:52MH:40Mf:82ff

^a^Data are referred only to individuals for which both phenotypic and genotypic information could be obtained, thus excluding those with missing data

The majority of the cultivars grown for table grapes and wine production in the germplasm population evaluated for the sex phenotype produced self-fertile hermaphroditic flowers (168) and three bore female flowers (Dattier noir, Picolit and Moscato Rosa), whereas *sylvestris* was dioecious with 46 % male and 54 % female individuals.

**Fig. 2:**
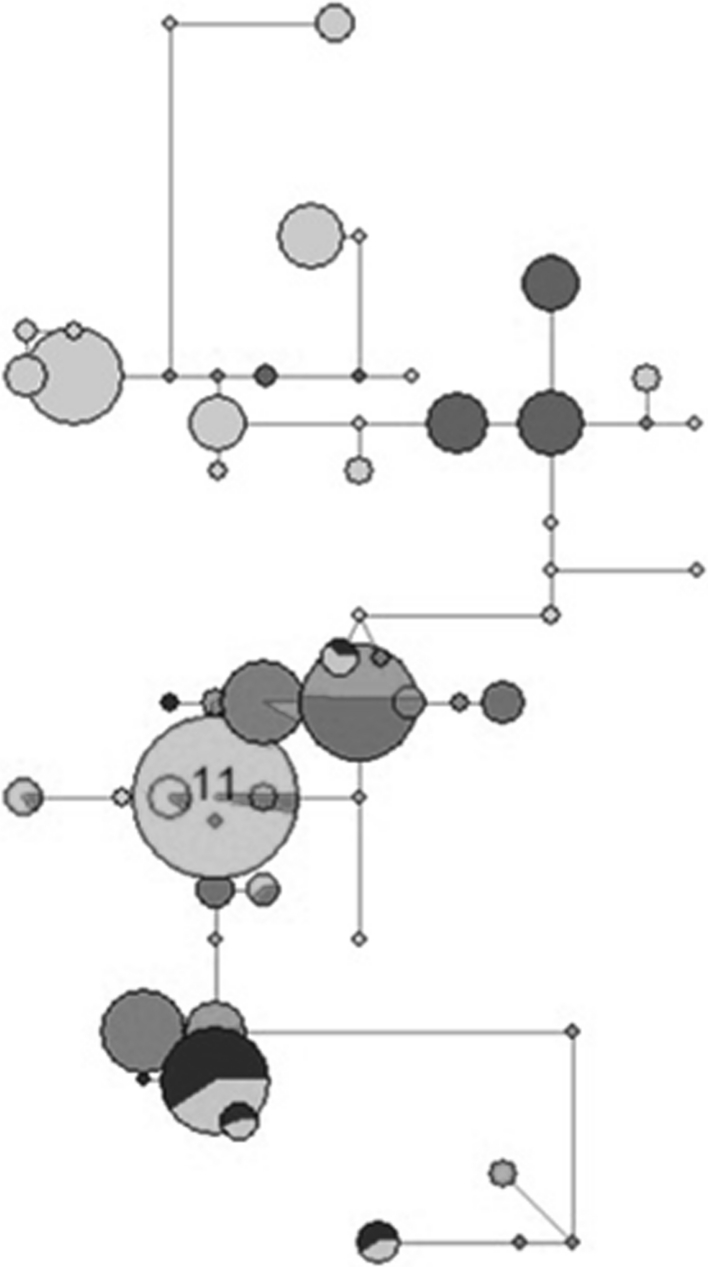
Median-joining networks derived from 55 reconstructed haplotypes capturing the sex locus based on three SSR markers. *Circles* represent distinct haplotypes and are scaled to reflect their frequencies. The branches connecting haplotypes indicate the mutational step between them. *Blue* haplotypes carrying the 304 bp allele at locus VVIB23; *light-green* haplotypes found in *V. sylvestris* accessions carrying the 288 bp allele at locus VVIB23; *orange* haplotypes found in *V. vinifera* cultivars carrying the 288 bp allele at locus VVIB23; *purple* haplotypes found in *V. vinifera* cultivars carrying the 284 bp allele at locus VVIB23; *dark*-*green* haplotypes found in *V. sylvestris* accessions carrying the 290 bp allele at locus VVIB23; *yellow* haplotypes found in *V. vinifera* cultivars carrying the 290 bp allele at locus VVIB23. *11* haplotype 11 carrying the 284 bp allele at locus VVIB23 (Color figure online)

Rather extensive (5–10 cM) linkage disequilibrium (LD) mainly of haplotypic origin has been reported between SSR loci for cultivated grapevine as a result of hermaphroditism selection during grape domestication followed by vegetative propagation [24]. However, a rapid LD decay was measured from SNP genotype data in *V. vinifera* [25], even if a resulting increase in LD might be seen for traits that experienced significant selection. This was shown for the berry pigmentation locus [25] which maps to the opposite side of chromosome 2 with respect to the sex locus. In our study, allele distribution and relationships between three SSR markers were determined in 300 accessions of *V. vinifera* for the chromosome region capturing the sex locus in both molecular maps (Fig. S1). A total of 55 reconstructed haplotypes based on VMC6F1, VVIB23 and VVMD34 markers were observed and used in the MJ network analysis (Fig. 2). Haplotypes carrying the VVIB23 allele fragments of 284, 288 and 290 bp were found to be closely interconnected whereas those carrying the 304 bp allele resulted somehow distinct. The 304 bp fragment was found almost exclusively in *sylvestris* males (60/61) and always in the heterozygous state. The majority of the American rootstocks (12/15), instead, displayed a 306 bp allele. Given that these sizes were significantly associated with the *M* allele, the haplotype networks suggest that the sequence variation conjectured to be responsible for the appearance of hermaphrodite flowers [1] may have occurred on the *f* allele rather than on the *M* allele in dioecious wild plants.

The analysis showed the haplotype 11 (alleles VMC6F1-136, VVIB23-284 and VMD34-239) as the most prominent from all the reconstructed haplotypes. This accounted for 66 % of the hermaphrodite and 2 % of the *sylvestris* accessions (Table 3) and may be evidence for a selection favouring plant productivity during domestication and post-domestication. However, the presence of a major haplotype at the sex locus in *vinifera* cultivars may also be explained by the relative small number of elite genotypes used in grape breeding that characterize the complex genetic structure of cultivated grapevine [25]. Homozygosity of haplotype 11 across all three loci was found in only 8 % of the hermaphrodite cultivars comparable with the low frequency of *HH* genotypes reported by Fechter et al. [11] for a single marker linked to the sex locus.

**Table 3:** Marker trait association in cultivated and wild *Vitis vinifera* germplasm

Fragment size of VVIB23 marker (bp)^a^	*V. vinifera*	*V. sylvestris*		Association test (logistic regression)	
	*H* (168)	f (71)	*M* (61)	*V. vinifera* (H) vs *V. sylvestris* (M or f)	*V. sylvestris*
		(49)^b^	(98)^b^		(M vs f)
284	128 [18/110]	9 [0/9]	4 [0/4]	*P* < 0.0001	ns
		6 [0/6]^b^	2 [0/2]^b^	NA	NA
288	49 [6/43]	56 [34/22]	37 [0/37]	*P* < 0.0001	*P* < 0.0001
		46 [17/29]^b^	73 [0/73]^b^	NA	NA
290	45 [1/44]	17 [4/13]	11 [0/11]	ns	ns
		13 [0/13]^b^	16 [0/16]^b^	NA	NA
302	32 [1/31]	3 [1/2]	2 [0/2]	ns	ns
		0 [0/0]^b^	12 [0/12]^b^	NA	NA
304	1 [0/1]	1 [0/1]	60 [0/60]	*P* < 0.0001	*P* < 0.0001
		1 [0/1]^b^	94 [0/94]^b^	NA	NA
310	55 [0/55]	12 [1/11]	5 [0/5]	ns	ns
		5 [0/5]^b^	3 [0/3]^b^	NA	NA
Haplotype 11	111 [9/102]	2 [0/2]	1 [0/1]	*P* < 0.0001	ns

Number of hermaphrodite (*H*), female (*f*) and male (*M*) accessions carrying the allele at VVIB23 locus or haplotype 11 in [homozygous/heterozygous] state are reported as well as significance of association tests (*P* values are Bonferroni-corrected)

*ns* Not statistically significant, *NA* not analyzed

^a^Fragment sizes of over 2 % frequency were considered

^b^Wild grapevine accessions recently collected and used to test the diagnostic potential of VVIB23 marker

Alleles 284, 288 and 304 bp at the VVIB23 locus were found to be statistically significantly associated with the sex alleles *H*, *f* and *M*, respectively, despite the 284 bp fragment is present in some *V. sylvestris* individuals and the 304 bp fragment was found in two (non-male) exceptions (Table 3).

The fact that a 288 bp fragment was found to co-segregate with the *H* sex allele in ‘Malvasia di Candia’ may be just the result of a recombination event happened in its hermaphrodite relatives, since association of this marker size with the female allele co-occurred with homozygosity in a good half of the female plants. Similar to ‘Malvasia di Candia’, genotype 288–290 bp was observed in 9 hermaphrodite cultivars and even 6 other hermaphrodites were homozygous 288–288 bp. The female accessions, Dattier noir (290–290 bp), Picolit (288–290 bp) and Moscato Rosa (290–302 bp), displayed genotypes that would support the relationship between a 290 bp fragment at VVIB23 locus and the sex allele *f* in cultivated varieties, although the association shown in Moscato Ottonel × Malvasia di Candia was not statistically significant in the germplasm. On the other hand, the occurrence in Moscato Rosa of a VVIB23 fragment (302 bp) co-segregating with the *M* sex allele in *V. riparia* seems contradictory, though the low frequency of this allele size discourage interpretation. Moreover, the extent of allele size homoplasy, for instance caused by insertions/deletions in SSR flanking regions, was not investigated in this study.

The diagnostic potential of VVIB23 marker was further proved in 147 non-redundant genotypes of *V.* *sylvestris* recently collected in the Italian Peninsula [26]. Consistent with previous findings, the 304 bp allele was found in 94 out of the 98 vines bearing male flowers, while the 288 bp allele was found in 46 out of the 49 female accessions (Table 3). The *H* linked fragment (284 bp) was detected in a few wild accessions and this may indicate a degree of misidentification in the sample or hybridization between wild male and cultivated forms as a result of pollen flux [27]. Given that *V. sylvestris* is still considered as a gene pool for viticulture, allelic diversity at VVIB23 locus may be exploited both for defining the status of wild germplasm and for integrating it on marker-assisted breeding programmes.

Although the gene mechanism governing sex determination in grapevine is yet to be elucidated, this work provides further mapping information and initial evidence that domestication may have affected haplotype frequency in the major candidate region for the sex phenotype.

## Electronic supplementary material

Below is the link to the electronic supplementary material.

Supplementary material 1 (DOC 103 kb)
