# Supplementary material for: Linkage Mapping and Molecular Diversity at the Flower Sex Locus in Wild and Cultivated Grapevine Reveal a Prominent SSR Haplotype in Hermaphrodite Plants
Source: Mol Biotechnol. 2013 Mar 27;54(3):1031–7. doi: 10.1007/s12033-013-9657-5 (PMC3641292; doi:10.1007/s12033-013-9657-5)
Supplement: Supplementary file 1 — Supplementary material 1 (DOC 103 kb) [file 12033_2013_9657_MOESM1_ESM.doc]

**SUPPLEMENTARY MATERIALS**

**Table S1**. Genes predicted from the reference sequence (PN40024 12 x version) in the region of interest for the sex locus between SNP4C_1 and VVIB23 markers.

|  |  |  |  |  |  |
| --- | --- | --- | --- | --- | --- |

| **Gene ID** | **Start** | **End** | **Strand** | **Annotation NCBI** |
| --- | --- | --- | --- | --- |
| LOC100260606_gene | 4666161 | 4667903 | + | pentatricopeptide repeat-containing protein At4g22760-like |
| LOC100265823_gene | 4671550 | 4672912 | + | unknown |
| LOC100243038_gene | 4685949 | 4693209 | - | deoxyribodipyrimidine photo-lyase-like |
| LOC100253287_gene | 4701544 | 4703868 | - | unknown |
| LOC100260132_gene | 4716742 | 4718407 | - | unknown |
| LOC100243132_gene | 4732422 | 4736741 | + | unknown |
| LOC100265433_gene | 4737981 | 4745369 | + | peptidyl-tRNA hydrolase ICT1, mitochondrial-like |
| LOC100249883_gene | 4745673 | 4746494 | - | unknown |
| LOC100244756_gene | 4748421 | 4755341 | - | LRR receptor-like serine/threonine-protein kinase |
| LOC100232874_gene | 4762024 | 4762800 | + | serine/threonine kinase-like |
| LOC100249980_gene | 4768698 | 4781916 | - | maspardin-like |
| LOC100261883_gene | 4781893 | 4788854 | + | maspardin-like |
| LOC100854222_gene | 4802451 | 4813207 | - | UDP-glucuronate 4-epimerase 3-like |
| LOC100255700_gene | 4822433 | 4823312 | - | unknown |
| LOC100242025_gene | 4836974 | 4844953 | + | NAC domain-containing protein 7-like |
| LOC100250522_gene | 4845894 | 4855934 | - | unknown |
| LOC100267708_gene | 4862062 | 4864777 | + | axial regulator YABBY 1-like |

**Table S2.** Segregation of SSR markers and sex phenotype in two mapping populations. In Moscato Bianco x *V. riparia*, genotypes **aa** have male flowers while genotypes **ab** have female (100) and hermaphrodite (94) flowers. A, B, C and D alleles at VVIB23 locus were sized 290, 284, 302 and 288 base pairs respectively. In Moscato Ottonel x Malvasia di Candia, genotypes **aa** have female flowers while genotypes **ab, ac** and **bc** are hermaphrodites. A, B and C alleles at VVIB23 locus were sized 290, 284 and 288 base pairs respectively.

**Fig. S1.** Frequency distribution of allele sizes at VVMD34, VVIB23 and VMC6F1 microsatellite loci observed in 168 *V. vinifera* cultivars with hermaphrodite flowers, 71 *V. sylvestris* with female flowers and 61 *V. sylvestris* with male flowers. The frequency of each allele size was calculated on the assumption that homozygous genotypes carry two copies of the same fragment, hence the total number of alleles was twice the number of individuals.

Reference for allele sizes: Pinot Noir VVMD34 239/239; VVIB23 288/310; VMC6F1 130/142.
